# Severe Immune Checkpoint Inhibitor Hepatitis in *KRAS* G12C-Mutant NSCLC Potentially Triggered by Sotorasib: Case Report

**DOI:** 10.1016/j.jtocrr.2021.100213

**Published:** 2021-08-02

**Authors:** Parvin Begum, Robert D. Goldin, Lucia A. Possamai, Sanjay Popat

**Affiliations:** a Lung Unit, Royal Marsden NHS Foundation Trust, London, United Kingdom; b Department of Metabolism, Digestion & Reproduction, Imperial College London, London, United Kingdom; c Thoracic Oncology, The Institute of Cancer Research, London, United Kingdom

**Keywords:** Sotorasib, Autoimmune hepatitis, Immune checkpoint inhibitor, *KRAS* G12C, Case report

## Abstract

Sotorasib is a first-in-class small molecule that irreversibly inhibits KRAS G12C, locking it in an inactive state, inhibiting oncogenic signaling, and inducing a proinflammatory microenvironment. Here, we report the first case of life-threatening hepatitis in a patient with NSCLC shortly after commencing sotorasib, in which biopsy result was consistent with checkpoint inhibitor (CPI) immune-related adverse event, implicating sotorasib as being able to trigger CPI immune hepatitis. Given the large proportion of patients potentially treatable with sequential sotorasib after CPI, coupled with limited trial data, sotorasib-triggered CPI immune-related hepatitis should be considered in patients with sotorasib hepatotoxicity.

## Introduction

Sotorasib (AMG-510) is an irreversible small molecule inhibitor targeting the P2 pocket of KRAS G12C, present only in the guanosine diphosphate-bound form of the protein, thereby trapping it in the inactive state. The CodeBreak100 phase 1 to 2 trial in relapsed *KRAS* G12C-mutant advanced NSCLC identified 960 mg once daily as the recommended phase 2 dose. Serious treatment-emergent adverse event (AE) grade greater than or equal to 3 occurred in 46% with 22% fatality rate,^1^ although after causality attribution, the treatment-related serious treatment-emergent AE grade greater than or equal to 3 rate was 1.7% with no fatalities reported.^1^^,^^2^ In the registrational phase 2 part of the trial, 81% of 126 patients had previous checkpoint inhibitor (CPI) exposure and the treatment-related grade greater than or equal to 3 alanine transaminase (ALT) and aspartate transaminase rates were 6.3% and 5.6%, respectively, with no grade greater than or equal to 3 bilirubin rise.

## Case Presentation

**Figure 1:**
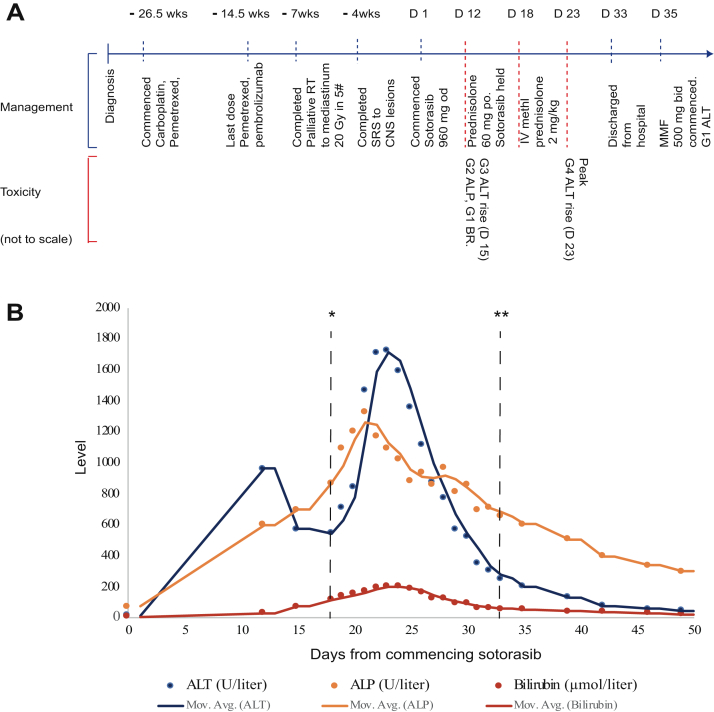
Patient timeline and liver function tests after commencement of sotorasib. (*A*) Schematic diagram revealing the timeline for the patient, from diagnosis to resolution of liver toxicity (not to scale). (*B*) Graph of liver function tests after commencement of sotorasib 960 mg QD, revealing exact values and average trend. A total of 60 mg oral prednisolone was started on D 15. High-dose IV methylprednisolone 2 mg/kg was started on D 19 and steroid wean initiated on D 31. ∗D of admission to hospital (D 18). ∗∗D of discharge from hospital (D 33). #, fractions of radiotherapy; ALP, alkaline phosphatase; ALT, alanine transferase; bid, twice daily; BR, bilirubin; CNS, central nervous system; D, day; G, grade; Gy, Gray; IV, intravenous; MMF, mycophenolate mofetil; Mov.Avg., moving average for value of each liver function test; po, orally; od, once daily; RT, radiotherapy; SRS, stereotactic radiosurgery; wk, week.

**Figure 2:**
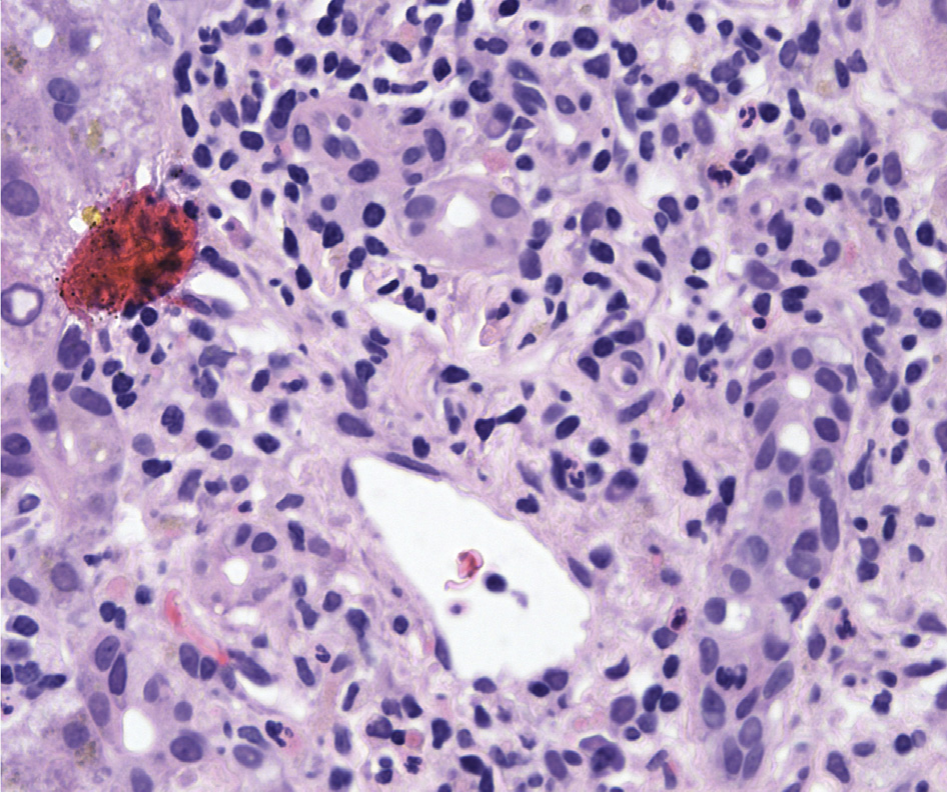
Representative section from liver biopsy indicating portal and lobular inflammatory changes consistent with CPI hepatitis. Portal tract with a mixed inflammatory infiltrate associated with bile duct damage (hematoxylin and eosin–stained section, original magnification ×400). CPI, checkpoint inhibitor.

A 62-year-old man was diagnosed in 2020 with having T2N3M1b adenocarcinoma subtype NSCLC, *KRAS* G12C mutant, and programmed death-ligand 1 positive at 2%. He had no relevant previous comorbidities, no previous history of liver disease, hepatitis, or alcohol abuse, and was positive for having cytomegalovirus immunoglobulin G. He was initially treated with carboplatin-pemetrexed-pembrolizumab, with mediastinal progression after cycle 1 of maintenance pembrolizumab-pemetrexed, for which he received palliative thoracic radiotherapy (20 Gy in 5 fractions). CPI therapy had caused no previous hepatotoxicity and no immune-related AEs worse than grade 1. He commenced second-line sotorasib, 960 mg once daily, on compassionate supply prelicense in March 2021, 14 weeks after the last dose of pembrolizumab, having discussed risks and alternatives. Baseline liver function tests before commencing sotorasib were within the reference range. On cycle 1 day 12, he had grade 2 alkaline phosphatase (ALP), grade 1 bilirubin, and grade 3 ALT rise. Sotorasib was held. A timeline of his treatment and AEs is outlined in Figure 1*A.* His bilirubin, ALT, and ALP worsened rapidly in subsequent days (Fig. 1*B*), requiring commencement of 60 mg oral prednisolone on day 15 and hospitalization. Investigations including liver ultrasonography, magnetic resonance cholangiopancreatography, and comprehensive acute viral serologic and molecular testing identified no other hepatotoxicity causes, including extensive testing for hepatitis A/B/C/E and a broad autoimmune antibody panel. Other than a positive Epstein–Barr virus immunoglobulin G and detection at low-level with polymerase chain reaction (346 IU/mL), considered noncontributory to this extreme hepatitis picture, no other notable inciting factors were present in the clinical and pharmacologic history or identified through investigations. Liver function continued to deteriorate. He was empirically treated for presumed immunotherapy-related hepatitis with 2 mg/kg intravenous methylprednisolone at day 19 and N-acetylcysteine support. At peak, his ALT reached 23.9 times the upper limit of normal (ULN) (ALT = 1722 U/liter, grade 4, d 23), 10.5 times the ULN ALP (1326 U/liter, grade 3, d 21), 9.3 times the ULN bilirubin (205 μmol/liter, grade 3, d 24), and 29.2 times the ULN gamma-glutamyl transferase (2131 U/liter, grade 4). Coagulation was not markedly deranged throughout (peak international normalized ratio = 1.2), and he did not develop encephalopathy. Ultrasound-guided liver biopsy (d 21) result identified portal and lobular inflammatory changes in a pattern typical of CPI hepatitis on expert review (Fig. 2). There was evidence of marked hepatocyte regeneration and significant cholestasis. Liver function tests improved by day 31, and a gradual steroid wean was initiated with ALT resolving to grade 1 by day 35.

## Discussion

*KRAS* G12C-mutant NSCLC is an aggressive disease associated with an immunosuppressive microenvironment. In preclinical studies, sotorasib resulted in a proinflammatory tumor microenvironment with durable responses alone and in combination with CPIs. The CodeBreak100 trial identified low rates of grade greater than or equal to 3 treatment-related ALT and aspartate transaminase elevation in phase 1 or grade greater than or equal to 3 bilirubin rise,^2^ and there was no marked additional excess of treatment-emergent hepatotoxicity. Grade 3 hepatitis was noted in one patient (0.8%) in the phase 1 portion of the trial but with no note of a biopsy result to confirm etiology. There were two cases of grade 3 (1.6%) drug-induced liver injury in the CODEBREAK 100 phase 2 trial, again with no information regarding whether a biopsy was undertaken to elucidate the exact mechanism and consider CPI-induced hepatitis as a differential. Preclinical studies have revealed that sotorasib rendered a proinflammatory tumor microenvironment highly sensitive to immunotherapy. It is therefore important to consider whether this may also potentiate CPI-related toxicities in those in which immunotherapy agents formed part of previous line treatments, although in this case we cannot fully exclude hepatitis occurring out with sotorasib exposure. Currently, impact of sotorasib toxicities by previous CPI use is unknown and remains an important question, because most sotorasib-eligible patients will be CPI exposed, and combination sotorasib-CPI data are lacking. Our case therefore represents an unexpected high-grade hepatic toxicity. The etiology of sotorasib-associated hepatocellular injury has not been identified to date, considered to be direct drug hepatotoxicity and mostly manageable with treatment hold and dose reduction. Nevertheless, our case confirmed CPI-mediated, immune-related hepatitis consistent with the hypothesis that in some CPI-exposed patients, sotorasib may induce a proinflammatory state sufficient to trigger immune-related hepatitis. This is consistent with emerging evidence for CPI-mediated, immune-related AEs requiring a priming event, with established triggers including small molecule drugs osimertinib^3^ and selpercatinib^4^ and cytomegalovirus reactivation.^5^ We therefore identify for the first time that sotorasib may trigger CPI-mediated, immune-related hepatitis in an anti–programmed cell death protein-1 immunotherapy exposed patient. It is unclear if this is a drug or class-specific effect.

## Conclusion

CPI-mediated, immune-related hepatitis triggered by sotorasib requiring steroid immunosuppression, not just drug hold should be considered in patients with hepatotoxicity.

## CRediT Authorship Contribution Statement

**Sanjay Popat:** Conceptualization ideas, Review and editing of first and final drafts, Coordinated patient’s care.

**Parvin Begum:** Writing original draft, Created Figure 1, Review and editing.

**Robert D. Goldin:** Histopathology review, Provided histology image, Review and editing.

**Lucia A. Possamai:** Specialist hepatology input, Review and editing.
